# Highly pathogenic avian influenza A virus (H5N1) can be transmitted in ferrets by transfusion

**DOI:** 10.1186/1471-2334-14-192

**Published:** 2014-04-08

**Authors:** Xue Wang, Jiying Tan, Jiangqin Zhao, Zhiping Ye, Indira Hewlett

**Affiliations:** 1 Lab of Molecular Virology, Building 29B, Rm 4NN22, Division of Emerging and Transfusion Transmitted Diseases, CBER/FDA, 8800 Rockville Pike, Bethesda, MD 20892, USA; 2 Division of Viral Products, Center for Biologics Evaluation and Research, Food and Drug Administration, Bethesda, MD 20892, USA

**Keywords:** H5N1, Transfusion, Viremia, Ferrets

## Abstract

**Abstracts:**

**Background**

Highly pathogenic avian influenza A virus has been shown to infect organs other than the lung, and this is likely to be mediated by systemic spread resulting from viremia which has been detected in blood in severe cases of infection with avian H5N1 viruses. The infectivity of virus in blood and the potential for virus transmission by transfusion has not been investigated.

**Methods**

Using a susceptible ferret animal model, we evaluated viremia and transmission by blood transfusion. Blood was collected on day 2, 4, 6, and 10 post-infection (or before death) from donor ferrets infected with either low dose (1.0 × 10^2.6^ EID_50_/ml) or high dose (1.0 × 10^3.6^ EID_50_/ml) of H5N1 virus, A/VN/1203/04 and transfused to recipient animals.

**Results**

Viremia was observed in 2/12 (16.67%) recipients that received blood from donor ferrets infected with low dose and 7/12 (58.33%) recipients who received blood from high dose infected donors. 1/12 (8.3%) low dose recipients and 6/12 (50%) high dose recipients died within 11 days after transfusion. Increased changes in body weight and temperatures were observed in high dose recipients, and high levels of viral RNA were detected in recipient ferrets after transfusion of blood from the early viremic phase, which also correlated with adverse impact on their survival.

**Conclusion**

These data suggest that highly pathogenic avian influenza A virus, H5N1, is transmissible by blood transfusion in ferrets. Low levels of viremia were detected around the time of onset of symptoms and later in ferrets infected with highly pathogenic H5N1 virus. These findings may have implication for pathogenesis and transmissibility of H5N1.

## Background

Influenza A viruses belong to the *Orthomyxoviridae* family of RNA viruses that contain eight segments of negative sense RNA [1]. There are several subtypes, numbered according to antigen HA and NA, containing sixteen different HA antigens (H1 to H16) and nine different NA antigens (N1 to N9) have been identified to date. Influenza A viruses evolve with high mutation rates and can occasionally cross the species barrier. Highly pathogenic avian influenza virus H5N1 originated in poultry and has been occasionally transmitted to humans resulting in high mortality [2]. There have been no reports thus far to indicate that H5N1 is readily transmissible from human to human.

H5N1 virus continues to circulate among poultry in many countries in Asia, Africa, and Europe, occasionally spreading to humans. In 1997, avian influenza A virus subtype H5N1 emerged capable of infecting humans with a highly fatal disease outcome (fatality rate of ~60%) [3,4]. The primary pathologic process that causes death is fulminant viral pneumonia [3,5]. High replication efficiency, broad tissue tropism and systemic replication seem to determine the pathogenicity of H5N1 viruses in animals [2,6]. A human isolate A/Vietnam/1203/04 (H5N1) was reported to be highly pathogenic and the severity of disease was associated with broad tissue tropism and high virus titers in multiple organs, including the brain in ferrets [6].

Some studies have shown that H5N1 virus is found exclusively in the respiratory tract (mainly in the lung) [7,8]. Other studies report the presence of H5N1 viruses in many extrapulmonary organs, such as intestine, liver, and brain [9-14]. Viral RNA has been detected in nasopharyngeal aspirates ranging from 1 day up to 15 days after disease onset [15,16]. Viral replication appears to be prolonged in H5N1 influenza because viral loads when plotted against time did not show a clear decline in a large group of H5N1 patients [17]. These findings demonstrate that H5N1 can be detected in brain, intestine, liver, lymph nodes, placenta, and fetal lung and cause coma, diarrhea, and encephalopathy in children [18], in addition to pneumonia.

Early in the 1960s, viremia was found in patients infected with influenza A virus in Asia [11,12]. Since then, other groups have reported that influenza viral RNA could be detected in blood from infected patients [17,19-22]. It has also been reported that detectable viral RNA in the blood of humans was associated with fatal outcomes while no viral RNA was detected in the blood of surviving H5N1-infected individuals [17].

Ferrets are an excellent mammalian animal model for studies of influenza virus pathogenicity and host immunity, and disease manifestations of influenza virus infection in ferrets closely resemble those in humans [6]. Recently, we found that infection with a human isolate, A/Vietnam/1203/04 (H5N1), resulted in viremia in the ferret model, which positively correlated with animal death [6]. Viral RNA could also be detected in brain, lung, ileum, nasal turbinate, and nasal wash. Although influenza viruses in blood and plasma are very stable [23], it is unclear whether the influenza virus can be transmitted by blood transfusion. Here, we performed studies in ferrets infected with the H5N1 strain to determine whether influenza A virus can be transmitted through blood transfusion.

## Methods

### Virus and its titer determination

A H5N1 strain, A/Vietnam/1203/04, was obtained from the Centers for Disease Control and Prevention (Atlanta, GA) (CDC#2004706280, E1/E3 (1/19/07) and amplified in 10–day old embryonated hen’s eggs (CBT Farms, Chestertown, MD). The virus was maintained at −80°C until use. For infectious titer determination 10-fold dilutions of virus stock was inoculated into 10-day old embryonated hen’s eggs (4–8 eggs per dilution) and the eggs were incubated for 48 hours. 50 μl of allantoic fluid was then collected from each egg and added to a microtiter plate. 50 μl of 0.5% turkey red blood cells (tRBCs) were added to all wells and plates were incubated for 30 minutes at room temperature. Plates were read for agglutination or non-agglutination. The 50% endpoint was determined by the method of Reed and Muench [24] from virus dilutions testing positive for hemagglutinin activity in tRBCs. Data were expressed as 50% egg infectious dose (EID_50_) per milliliter.

### Ferret inoculation and transfusion

Forty two adult male ferrets (Triple F Farms, Sayre, PA) that were 6 ~ 7 months of age and seronegative for representative currently circulating human influenza A strains and the challenge virus prior to initiation of the study were identified for the study. Ferrets were housed and cared for at BIOQUAL, Inc. (Rockville, MD). The BIOQUAL, Inc. IACUC had approved the animal care and use proposal, ACUP # 11-3056-71, prior to start of the study. For all procedures, the ferrets were lightly anesthetized with a solution of ketamine/xylazine formulated to provide doses of 25 mg/kg ketamine and 2.0 mg/kg xylazine to each animal. The animals (donors) were inoculated intranasally with 500 μl of virus, approximately 250 μl to each nare. Six donor ferrets were infected with low dose, about 1.0 × 10^2.6^ EID_50_/ml of virus and the other 6 donors were infected with high dose of about 1.0 × 10^3.6^ EID_50_/ml of virus. For each challenge dose, the donor animals were divided into two groups; blood was collected on days 2 and day 6 post-infection from one of these groups and on days 4 and day 10 post-infection from the other group. At these time points about 2 ml of freshly collected blood was transfused into its appropriate recipient (see Table 1 for details). To reduce the risk of a minor allergic reaction the recipient ferrets were administered a dose of antihistamine (Benadryl, 2 mg/kg, IM) 5 min. prior to the transfusion. A 24 gauge IV catheter was then inserted into the cephalic vein and a blood line with filter was connected. The anti-coagulated (acid citrate dextrose (ACD). 0.48% (w/v) citric acid. 1.32% (w/v) sodium citrate. 1.47% (w/v) glucose) blood from one donor was slowly pushed through the line over a 10–15 minute period. In total, 24 recipients received blood from H5N1-infected animals and 3 animals received blood from animals treated with equal volume of PBS in each nare as controls.

**Table 1:** Study design of donors and their appropriate recipients

**Donor group**	**Animal code**	**Day blood collected**	**Recipient code**
Low dose	51	2	63
		6	67
	52	2	64
		6	68
	53	2	66
		6	69
	54	4	70
		10	73
	55	4	71
		10	74
	56	4	72
		10	75
High dose	57	2	76
		6	79
	58	2	77
		6	80
	59	2	78
		6	81
	60	4	82
		10	85
	61	4	83
		10	86
	62	4	84
		10	87
PBS	49	4	88
	50	6	89
	62	10	90

Clinical signs of infection, weight, and temperatures were recorded twice daily. Activity scores were assigned as follows: 0, alert and playful; 1, alert but playful only when stimulated; 2, alert, but not playful when stimulated; and 3, neither alert nor playful when stimulated. Ferrets that showed signs of severe disease (prolonged fever; diarrhea; nasal discharge interfering with eating, drinking, or breathing; severe lethargy; or neurological signs) or had >20% weight loss were euthanized immediately. Euthanasia was performed on ferrets that had been sedated using IM inoculation with a ketamine HCl (25 mg/kg) and xylazine (2 mg/kg) solution. Blood was obtained from recipients on days 2, 4, and 8 post-transfusion (or before animal death) and analyzed for viral loads using RT-qPCR. These studies were performed in a Biosafety level 3 enhanced laboratory.

### Nasal wash

Nasal wash samples were collected from all ferrets on days 0, 2, 4, 6, and 8 for viral load determination. Briefly, the ferret was sedated with ketamine/xylazine and placed in the laminar flow hood, draped over a small box. With the nose pointed upwards, 3 ml PBS/gentamicin/0.5% BSA solution is slowly instilled into the nostrils using a 24 gauge ¾” plastic catheter connected to a 3 ml syringe. The nasal wash was collected into a 15 ml sterile conical tube, spun down to remove cell debris. 0.5 ml sample was used for immediate isolation of viral RNA, and an additional sample used for the TCID_50_ assay. These procedures were performed in a Biosafety level 3 enhanced laboratory.

### RT-qPCR

Quantitative real-time RT-PCR was used for detection of viral RNA in blood and nasal wash. 500 μl of blood or nasal wash were used to isolate nucleic acids by using the QIAamp Viral RNA Mini Kit (Valencia, CA 91355) according to the manufacturer’s protocol. We designed a set of primers and probes for the matrix gene, M, of the avian H5N1 influenza A virus, according to GenBank database. The forward primer was 5′-CGTCAGGCCCCCTCAAA-3′, and the reverse primer was 5′-GGTGTTCTTTCCTGCAAAGA-3′. The TaqMan probe was oligonucleotide 5′-TCAAGTTTCTGTGCGATCT-3′, coupled with a reporter dye [6-carboxy fluorescein] (FAM) at the 5′ end, a non-fluorescent quencher and a minor groove binder (MGB), that served as a Tm enhancer, at the 3′ end. The nucleic acids were amplified and detected in an automated TaqMan 7500 Analyzer by using QuantiTect™ Probe RT-PCR kit (Qiagen Inc., Valencia, CA). The 25-μl PCR mixture consisted of 100 nM primers and 100 nM probe. Following three thermal steps at 55°C for 5 min, at 50°C for 30 min and at 95°C for 10 min, 45 cycles of two-step PCR at 95°C for 15 s and at 60°C for 1 min were performed. The limit of detection was 1 fg of virus RNA per reaction with the TaqMan assay initial sample dilution at 1:10.

### TCID_50_ assay

The nasal wash and plasma samples were added in 10-fold graded dilutions to 96-well round-bottomed tissue culture plates. As a positive control, 10-fold dilutions of the challenge virus were included in each experiment. MDCK cells were then added to all wells and the plates incubated for 48 hr at 37°C, 5% CO_2_. Following this period 50 μL from each well was transferred to a 96-well V-bottomed microtiter plate, 0.5% turkey RBC added to all wells and the presence of virus detected by hemagglutination as a read-out. The 50% endpoint was determined by the method of Reed and Muench [17] from virus dilutions testing positive for hemagglutinin activity in Turkey Red Blood Cells (tRBC). Data were expressed as 50% tissue culture infective dose (TCID_50_) per milliliter.

### Statistical analysis

The log rank test was used for comparing survival curves and the unpaired Student’s *t* test was used for other data analyses as indicated, and a value of *p* < 0.05 was considered significant.

## Results

### Transfusion of viremic blood was associated with fatal outcome in recipients

**Figure 1:**
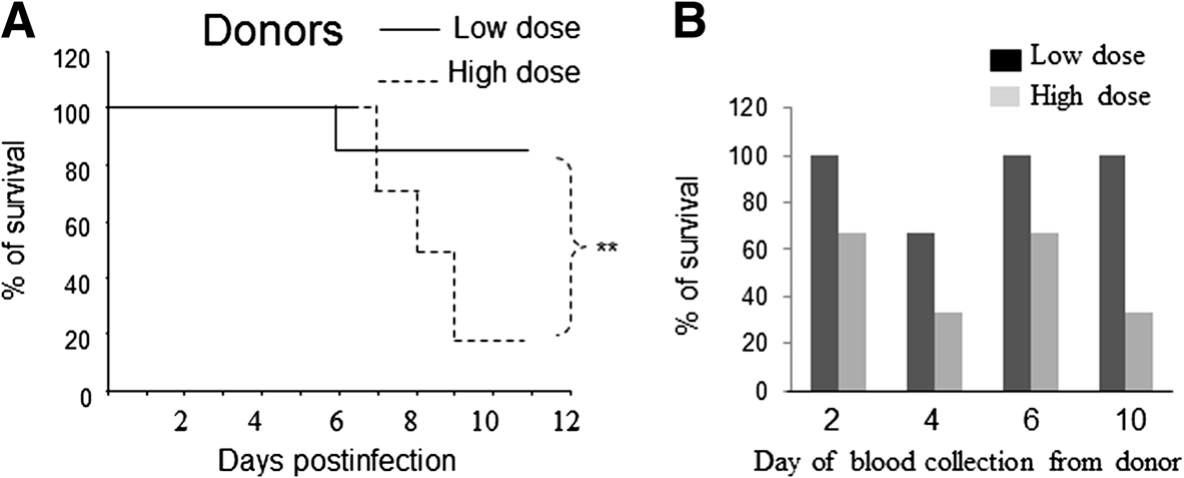
**Clinical signs of challenge with H5N1 virus A/VN/1203/04 in ferrets.** 6 ferrets (donors) were infected with 1 × 10^2.6^ EID50/ml (low dose) of virus and 6 other donors were infected with 1 × 10^3.6^ EID50/ml of virus. For each challenge dose, the donor animals were divided into two groups; blood was collected on days 2 and day 6 post-infection from one of these groups and on days 4 and day 10 post-infection (or the day before animal died) from the other group. At these time points about 2 ml of freshly collected blood was transfused into its appropriate recipient. **(A)**. Survival of ferrets after challenge. Ferrets (donors) were inoculated with either low dose or high dose of the virus and animal survival was recorded up to 12 days post-challenge. **(B)** Ferrets (recipients) were transfused with blood collected from donor animals on days 2, 4, 6, and 10 post-infection, or on the day the donor animal died; survival was recorded up to 12 days post-transfusion.

We infected 6 ferrets (donors) with 1.0 × 10 ^2.6^ EID_50_/ml (low dose) and another 6 animals with 1.0 × 10 ^3.6^ EID_50_/ml (high dose) of H5N1 virus, strain A/VN/1203/04. As shown in Figure 1A, mortality in donors occurred at a higher rate with high dose compared with low dose. Blood was collected from infected animals (donors) as scheduled (Table 1) or before the death of the animal and about 2 ml of the fresh blood was transfused to their appropriate recipients (Table 1). Fatal outcomes were observed in some recipient ferrets (Figure 1B). About 50% of ferrets died within 12 days post-transfusion of blood from ferrets that were infected with a high dose of virus compared with 10% of recipients from the low dose virus infected group (Figure 1B). All control animals remained healthy and symptom-free throughout the duration of the study. These data suggested that the fatal outcomes observed were likely to be associated with virus infection rather than the result of the transfusion procedure.

**Figure 2:**
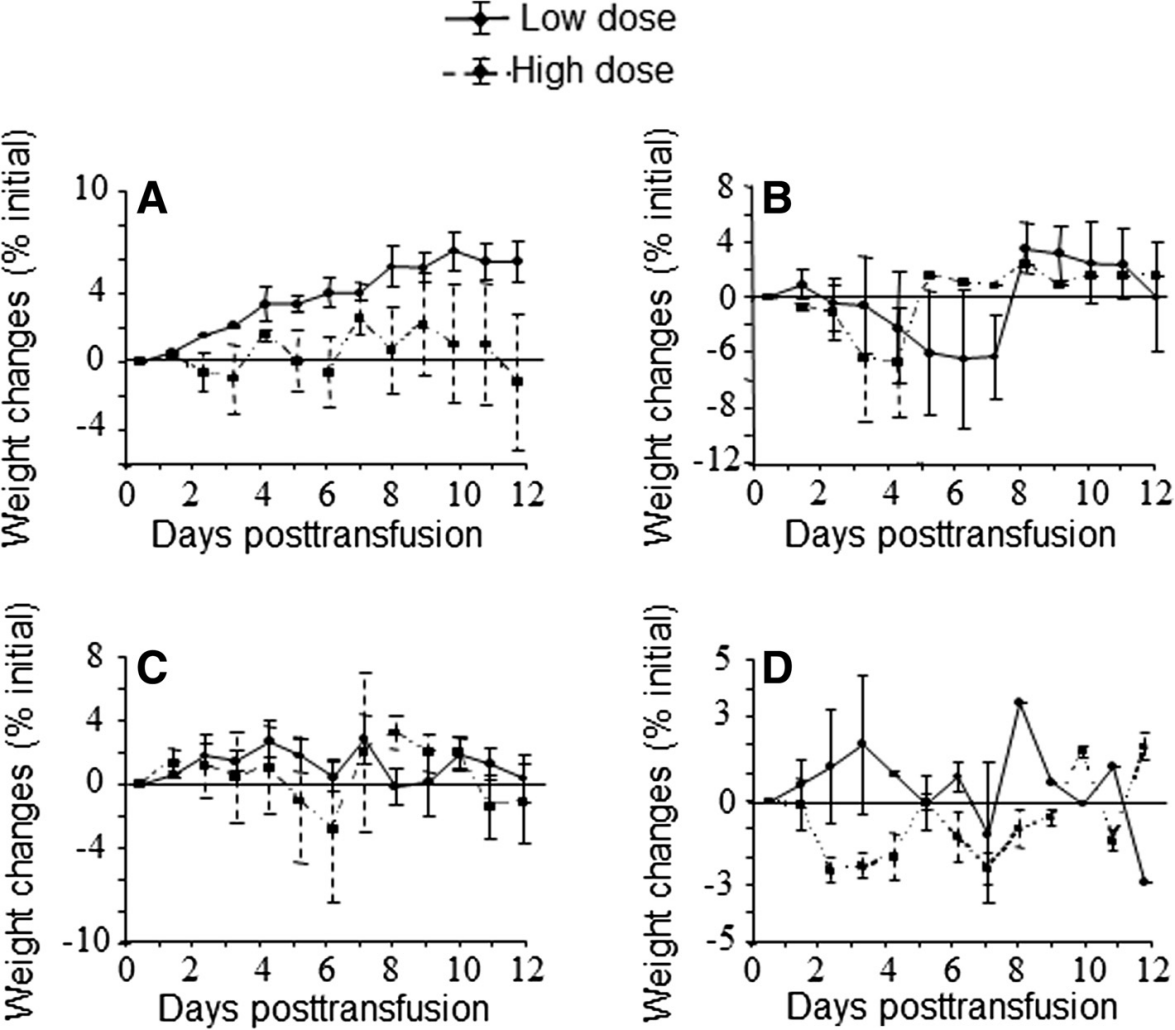
**Changes in weights of ferrets (recipients) transfused with blood from infected donor animals.** Ferrets (recipients) were transfused with blood from infected donor animals on the day post-infection when blood was collected, day 2 **(A)**, 4 **(B)**, 6 **(C)**, 10 **(D)**. The weights of ferrets were measured daily. The loss or gain of weight was calculated for each ferret as the percent change in the initial mean starting weight on day 0. Values are the averages ± SD for the ferret(s) alive for each group.

Changes in body weight (kg) and temperature (°C) were also recorded and calculated in Figure 2 and Table 2. In general, animals that received blood from ferrets infected with high dose of virus showed decreased body weight and increased body temperature relative to the recipients that received blood from ferrets infected with low dose of virus.

**Table 2:** Changes in body temperatures of recipients animals

**Day**	**Day 2**	**Collected**	**Day 4**	**Collected**	**Day 6**	**Collected**	**Day 10**	**Collected**
**Post- transfusion**	**Low dose**	**High dose**	**Low dose**	**High dose**	**Low dose**	**High dose**	**Low dose**	**High dose**
0	0.00 ± 0.76	0.00 ± 1.93	0.00 ± 1.73	0.00 ± 1.02	0.00 ± 0.96	0.00 ± 1.40	0.00 ± 0.91	0.00 ± 1.54
1	2.00 ± 1.04	2.54 ± 0.30	0.63 ± 1.13	3.50 ± 1.13	1.23 ± 0.47	1.50 ± 0.95	0.03 ± 1.51	−1.30 ± 0.29
2	0.83 ± 0.72	2.44 ± 2.00	1.43 ± 1.97	4.53 ± 1.45	1.13 ± 0.50	2.27 ± 2.44	1.00 ± 1.08	−0.13 ± 1.28
3	3.43 ± 0.44	4.44 ± 0.71	0.50 ± 2.19	2.93 ± 1.54	1.83 ± 0.25	2.60 ± 1.31	1.73 ± 0.65	−1.13 ± 2.18
4	2.06 ± 1.82	2.77 ± 2.83	−0.80 ± 1.80	−0.62 ± 1.20	1.97 ± 1.88	3.20 ± 1.81	−1.94 ± 0.95	−1.63 ± 3.25
5	1.10 ± 1.48	2.07 ± 0.00	−1.03 ± 0.98	4.73 ± 0.00	2.37 ± 0.76	2.90 ± 1.57	2.40 ± 0.55	0.82 ± 0.35
6	1.06 ± 0.78	0.02 ± 1.48	−0.73 ± 2.24	3.93 ± 0.00	2.97 ± 0.72	2.75 ± 2.76	0.36 ± 1.80	−1.93 ± 3.54
7	0.46 ± 0.68	3.02 ± 1.91	−1.05 ± 0.07	3.23 ± 0.00	0.77 ± 1.80	3.20 ± 2.40	1.83 ± 2.19	−0.88 ± 0.35
8	0.46 ± 1.16	1.37 ± 1.27	0.65 ± 1.34	3.43 ± 0.00	1.07 ± 0.35	2.50 ± 2.69	0.26 ± 0.91	0.07 ± 0.00
9	0.20 ± 1.22	1.92 ± 2.47	−0.60 ± 0.71	1.73 ± 0.00	1.27 ± 1.72	3.50 ± 2.55	1.53 ± 0.26	0.07 ± 0.00
10	0.30 ± 0.45	2.12 ± 1.91	−2.95 ± 1.63	3.83 ± 0.00	1.13 ± 0.71	2.40 ± 2.69	−1.64 ± 2.19	0.07 ± 0.00
11	1.20 ± 0.57	0.32 ± 2.90	1.30 ± 1.56	2.73 ± 0.00	2.90 ± 0.75	3.85 ± 2.47	−0.17 ± 1.77	0.07 ± 0.00
12	1.33 ± 2.19	−1.78 ± 0.64	−1.70 ± 0.57	−0.87 ± 0.00	−0.23 ± 1.22	2.10 ± 0.14	2.13 ± 0.69	0.07 ± 0.00
Average	1.11	1.77	−0.33	2.54	1.42	2.52	0.58	−0.71

Note: Ferrets (recipients) were transfused with blood from infected donor animals on the day when blood was collected (Day collected), and body temperatures were monitored daily by the use of subcutaneous implantable temperature transponders for 12 days post-transfusion. Each data point represents the mean value ± SD for the surviving ferrets.

### Viral RNA could be detected in the blood of both donors and recipients

Transfusion of blood from ferrets infected with high dose of virus resulted in the death of recipient ferrets which positively correlated with levels of viral RNA [6]. We measured viral RNA in ferret blood collected from donors on the day of transfusion, or from recipients on day 2, 4, 8 post-transfusion as scheduled or before the recipient died using the RT-qPCR assay. Viral RNA detection in blood from donors and/or their appropriate recipients are shown in Table 3. In the low dose group, viral RNA in blood could be detected only in one donor (1/6 ferrets) on day 10 post-infection; while 5/6 of donors displayed viral RNA in their blood in the high dose group. Partial identity of the viral matrix gene sequences with wild type sequence was found in the blood of some recipients, which were analyzed using cDNA sequencing (Additional file 1: Figure S1) which provided direct evidence of viremia.

**Table 3:** Viral load (fg/ml) in blood of ferrets (donors) infected with H5N1 virus, A/VN/1203/04 and the ferrets (recipients) transfused with blood from their appropriate donors using the RT-qPCR

**Donors**					**Recipients**			
**Group**	**Animal code**	**Day post- infection**	**Virus load (fg/ml) (mean ± SD)**	**Survival (days posttransfusion)**	**Animal code**	**Day post- challenge**	**Virus load (fg/ml) (mean ± SD)**	**Survival (days posttransfusion)**
Low dose	51	2	0^a^	>12	63	2,4,8	0	>12
		6	0	>12	67	2,4,8	0	>12
	52	2	0	>12	64	2,4,8	0	>12
		6	0	>12	68	2,4,8	0	>12
	53	2	0	>12	66	2,4,8	0	>12
		6	0	>12	69	2,4,8	0	>12
	54	4	0	>12	70	2	8.12 × 10^4^ ± 5.17 × 10^2^	6
						4	5.77 × 10^5^ ± 6.90 × 10^3^	
						6	4.02 × 10^6^ ± 3.94 × 10^5^	
		10	0	>12	73	2,4,8	0	>12
	55	4	0	>12	71	2,4,8	0	>12
		10	7.62 × 10^4^ ± 1.31 × 10^3^	>12	74	2,4,8	0	>12
	56	4	0	>12	72	2,4,8	0	>12
		10	0	>12	75	4	1.33 × 10^4^ ± 9.59 × 10^3^	>12
High dose	57	2	0	7	76	2,4,8	0	>12
		6	4.94 × 10^5^ ± 5.27 × 10^4^		79	8	2.11 × 10^4^ ± 4.23 × 10^3^	>12
	58	2	0	>12	77	2,4,8	0	>12
		6	0		80	2,4,8	0	>12
	59	2	7.62 × 10^4^ ± 3.57 × 10^3^	6	78	2	2.30 × 10^6^ ± 2.58 × 10^4^	4
		6	1.93 × 10^6^ ± 6.95 × 10^5^		81	2	2.01 × 10^4^ ± 1.60 × 10^3^	5
						4	8.59 × 10^4^ ± 5.63 × 10^3^	
	60	4	0	7	82	2	4.51 × 10^6^ ± 9.65 × 10^4^	4
		7	2.12 × 10^5^ ± 2.35 × 10^4^		85	2	5.81 × 10^5^ ± 3.65 × 10^4^	7
						4	1.18 × 10^7^ ± 5.99 × 10^5^	
	61	4	0	8	83	2,4,8	0	>12
		8	2.28 × 10^6^ ± 5.94 × 10^5^		86	4	4.79 × 10^4^ ± 2.54 × 10^3^	11
	62	4	5.10 × 10^4^ ± 2.37 × 10^3^	8	84	2	9.76 × 10^4^ ± 3.14 × 10^3^	5
						4	1.67 × 10^7^ ± 5.96 × 10^5^	
		8	1.33 × 10^4^ ± 6.59 × 10^2^		87	2,4,8	0	>12
PBS	49	4	0		88	8	0	>12
	50	6	0		89	8	0	>12
	62	10	0		90	8	0	>12

Note: a) 0 indicates that viral load could not be detectable in our experiment condition.

**Figure 3:**
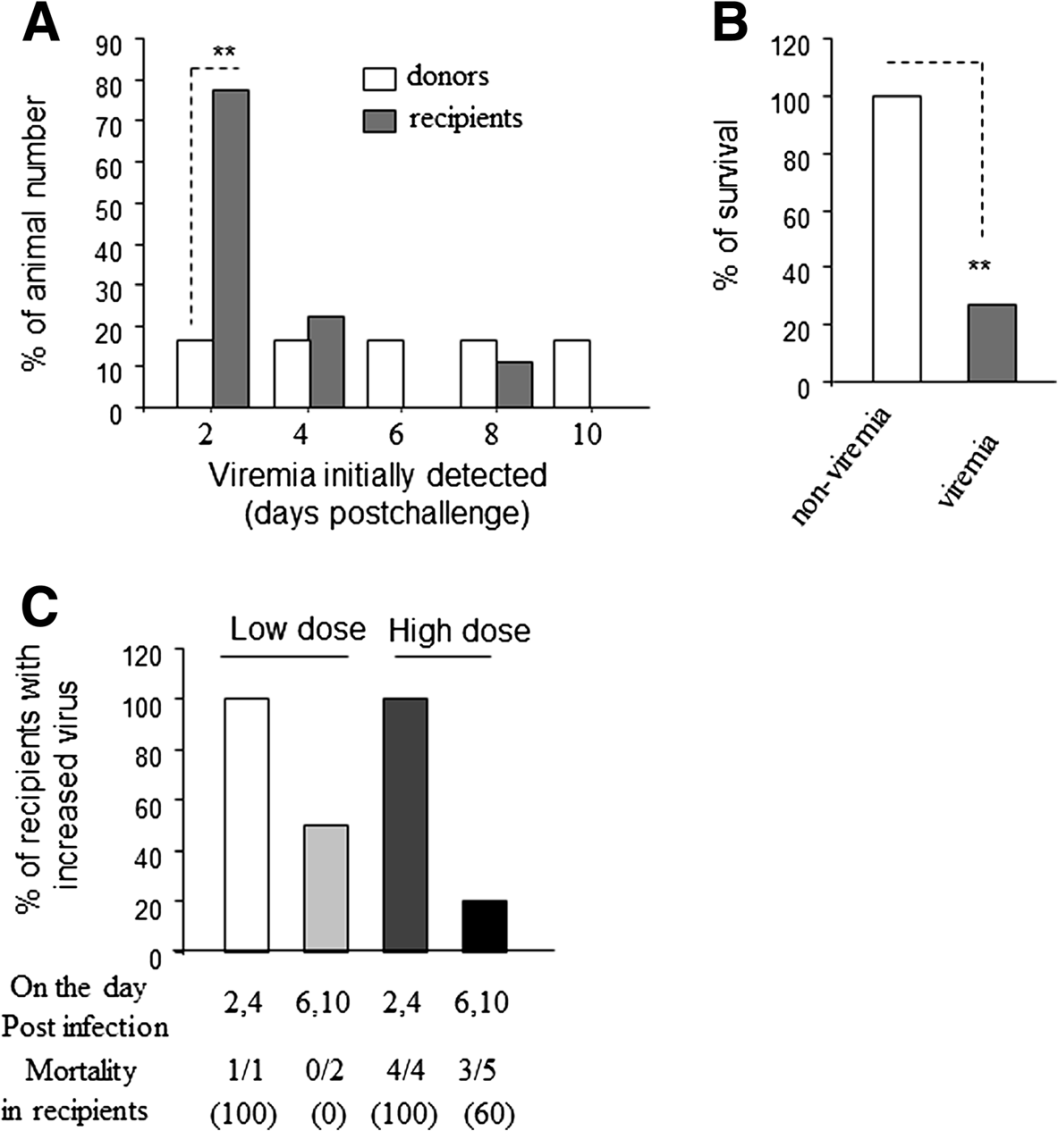
**Viremia and related clinical signs.** 6 ferrets (donors) were infected with 1 × 10^2.6^ EID50/ml (low dose) of virus and 6 other donors were infected with 1 × 10^3.6^ EID50/ml of virus. For each challenge dose, the donor animals were divided into two groups; blood was collected on days 2 and day 6 post-infection from one of these groups and on days 4 and day 10 post-infection (or the day before animal died) from the other group. At these time points about 2 ml of freshly collected blood was transfused into its appropriate recipient. **(A)**. Viremia is detectable in ferrets post-challenge. Virus RNA was isolated from blood collected from animals on day after post-infection (donors)/transfusion (recipients), virus loads were tested with RT-qPCR assay; and survival was recorded up to 12 days post-challenge. **(B)**. % survival of recipient ferrets with viremia vs. non-viremia. Recipients were followed up to 12 days post-transfusion; virus load was measured using an RT-qPCR assay. **(C)**. H5N1 replication in recipient ferrets. Viral loads in blood collected from recipients on day 2, day 4, day 6, and day10 post-transfusion, were detected using the RT-qPCR assay; animal survival was recorded up to 12 days after challenge.

Table 3 also showed that viral RNA could be detected in the blood of 3/12 (25%) ferrets that received blood from donors infected with low-dose of virus and 7/12 (58.33%) recipients that received blood from high-dose infected donors. These data indicate that highly pathogenic avian influenza A virus (H5N1) can be transmitted through blood transfusion. Most recipients had detectable viral RNA in blood collected on day 2 post-transfusion while donor animals showed viremia after day 2 post-infection (Figure 3A). We also found that most viremic recipients died while recipients without viremia had 100% survival (Figure 3B), indicating that viremia is associated with fatal outcomes in ferrets infected with highly pathogenic H5N1 influenza virus.

To understand the relationship of virus load in blood with animal death, we recorded animal death and detectable virus load in blood. As shown in Table 4, viral RNA levels greater than 8 × 10^4^ fg/ml in blood were associated with higher number of fatalities, while some recipients with viral RNA of more than 3 × 10^4^ fg/ml in blood also did not survive. These data indicate that transfusion of lower amount of virus load in blood could also result in recipient animal death, and that high titers of viremia are a strong predictor of fatality in the H5N1-infected host.

**Table 4:** The relationship between viral load (fg/ml) in blood and ferret death (days post-challenge)

**Group**	**Donors**			**Recipients**		
	**Animal code**	**Survival days**		**Animal code**	**Survival days**	
		**<8 × 10**^**4**^	**>8 × 10**^**4**^		**<3 × 10**^**4**^	**>3 × 10**^**4**^
Low dose	54	>12		70		6
	55	>12		74	>12	
	56	>12		75	>12	
High dose	57		7	79	>12	
	59		6	78		4
				81		5
	60		7	82		4
				85		7
	61		8	86		11
	62	8		84		5
				87	>12	

Note: data shown here only represented either animal of both donor and its appropriate recipient that viral RNA can be detectable in their blood under our experiment condition. And the animals with non-detectable (nd) viral RNA in blood did not show here.

### Higher levels of viral RNA were found in the blood of some recipients after transfusion of blood collected in the early viremic phase

Although viral RNA could not be detected in the blood of animal #54, #56 from the low dose group and animal #60 from the high dose group, viremia was detected in the blood of their recipients. Also some recipients given blood from the high dose group had higher levels of viral RNA than their donors on the day that blood was collected (Table 3). Figure 3C showed that 1/1 (100%), or 4/4 (100%) recipients of blood collected from donors on day 2 or day 4 post-infection displayed increased viral RNA in their blood (also see Table 3) after transfusion in both virus dose groups relative to their donors and mortality was 100%. Some recipients that survived did not show high amounts of viral RNA in their blood after transfusion of blood from donors at day 6 post-infection or later. These data indicated that higher titers of viral RNA could be detected in recipients of blood collected in the early viremic phase compared with the late viremic phase.

### Viral loads were detectable in nasal wash from recipients

Previously we reported that virus could be detected more frequently in the nasal washes of animals in the high dose group compared with those in the low dose group. Viral load was detected in nasal wash samples on day 2 in both groups [6]. In this study, we tested viral loads in the nasal wash from recipients and viral loads were detected in these samples (Table 5). 8/10 of recipients with viremia had detectable viral loads in the nasal wash, and two recipient animals (#81 and #79) with viremia did not have detectable virus in the nasal wash (compared Table 3 to Table 5). As shown in Table 5, there was a positive association of viral load measured using both RT-qPCR and TCID_50_ assays with lack of survival; generally detection of a higher viral load in nasal wash was associated with a decreased chance of recipient survival.

**Table 5:** **Viral loads assay in nasal wash from recipient ferrets that viral RNA could be detectable using either RT-qPCR or TCID**_**50 **_**assay**

**Group**	**Animal code**	**Day post- transfusion**	**Viral RNA (fg/ml) (mean ± SD)**	**lg (TCID**_**50**_**/ml)**	**Survival (days post-transfusion)**
Low dose	70	2	1.20 × 10^4^ ± 5.98 × 10^3^	<0.5	6
		6	1.36 × 10^7^ ± 4.60 × 10^5^	3.6	
	74	2	6.58 × 10^6^ ± 2.54 × 10^4^	0.5	>12
	75	13	1.63 × 10^4^ ± 3.92 × 10^3^	<0.5	>12
	69	14	2.01 × 10^4^ ± 2.24 × 10^3^	<0.5	>12
High dose	78	2	9.09 × 10^4^ ± 6.82 × 10^3^	1	4
	82	2	2.11 × 10^6^ ± 4.13 × 10^5^	1.5	4
	84	2	7.73 × 10^4^ ± 1.29 × 10^3^	<0.5	5
		4	6.53 × 10^6^ ± 5.43 × 10^5^	3.84	
	85	2	2.75 × 10^8^ ± 1.14 × 10^7^	4.84	7
		3	2.51 × 10^7^ ± 8.52 × 10^6^	0.5	
	86	2	2.72 × 10^4^ ± 2.45 × 10^3^	1.5	11
	87	2	1.32 × 10^4^ ± 5.97 × 10^3^	1.5	>12
	83	4	4.52 × 10^4^ ± 2.57 × 10^3^	<0.5	>12
	80	14	1.71 × 10^4^ ± 9.98 × 10^3^	<0.5	>12

Viral load testing using the RT-qPCR assay was more sensitive than the TCID_50_ assay. Twelve recipients displayed detectable viral loads in nasal wash using RT-qPCR assay, while only 8/12 of animals showed positive viral loads in the nasal wash (lg(TCID_50_/ml) >0.5) using TCID_50_ assay. These data indicate that H5N1 can be transmitted by transfusion of viremic blood and that transfusion resulted in increased virus levels in the recipient as evidenced by detectable viral load in the nasal wash of recipients using both RT-qPCR assay and TCID_50_ assays. High viral loads in blood and nasal wash of recipients correlated with fatal outcomes in these animals.

In summary, 4/12 recipients (33.33%) who received blood from donor ferrets infected with low dose displayed viral RNA in blood and/or nasal washes. 10 of 12 recipients (83.33%) who received blood from donor ferrets infected with high dose had detectable viral RNA in blood and/or nasal wash.

## Discussion

Although H5N1 infection of humans has been associated primarily with infection of the respiratory tract, dissemination of virus to other organs, such as the brain, has also been reported in several cases. Virus dissemination from lungs to extrapulmonary tissues most likely occurs by viremia. The isolation of highly pathogenic influenza H5N1 virus from the blood of 2 patients and the detection of viral RNA by RT-qPCR in the blood of 9 of 16 patients suggest that viremia can occur at high levels and for prolonged periods in people with symptoms of highly pathogenic influenza H5N1 virus infection [25]. Thus far, it has been shown that infection with both avian H5N1 virus [14,17] and pandemic H1N1 (swine) virus [22] can result in viremia, which is associated with severe disease manifestations and fatal outcomes.

Ferrets develop a productive infection after inoculation with human and avian influenza viruses without prior adaptation of the virus. Experimental influenza virus infections of ferrets have been used to model different aspects of influenza in humans [26]. The human isolate A/Vietnam/1203/04 (H5N1) is one of the most pathogenic virus isolates; and severity of disease was associated with broad tissue tropism and high virus titers in multiple organs, including the brain. High fever, weight loss, anorexia, extreme lethargy, and diarrhea were observed as major clinical signs and symptoms [6]. In addition, viral RNA is frequently detected in blood one or two days before death and viremia is associated with lethal outcomes [6].

Detection of virus in nasal washes is a key biomarker for influenza virus infection. In our study we showed that transfusion of viremic blood resulted in virus transmission in the recipient and that virus could be detected in nasal wash using RT-qPCR and TCID_50_ assays. Our study also showed that viremia correlated with detectable viral load in the nasal wash, and high amounts of virus in nasal wash as detected by the TCID_50_ assay was associated with recipient death (Tables 3 and 4).

Transfusion of blood collected on day 2 or day 4 post-infection resulted in higher levels of viral RNA in the blood of recipient ferrets and 100% fatality compared with levels observed in ferrets who received blood at from day 6 or later post-infection. One explanation may be that at the later periods of infection antibody production could have been initiated which would reduce potential for transmission by immune complex formation (Additional file 1: Table S1). However, these aspects of influenza transmission by viremic blood would need to be explored further.

The highly pathogenic influenza virus H5N1 has been shown to infect multiple human organs other than the lungs, suggesting that H5N1 can replicate in these organs. Quantitative RT-PCR showed that high viral load is associated with increased host responses [14]. High viral loads have been found in lung, brain and blood from ferrets infected with H5N1, A/VN/1203/04 and virus could replicate in these tissues and damage cells in these organs [6]. It has been reported that Influenza A virus can infect and replicate in T lymphocytes and peripheral blood mononuclear cells (PBMCs) [27], primary T cells, and Jurkat cells [28]. Currently, it is not known whether H5N1 viruses replicate in PBMCs and the type of cells in PBMCs that could support replication needs further investigation.

## Conclusions

In conclusion, our study has shown that highly pathogenic influenza A virus H5N1 can be transmitted through blood transfusion in a susceptible ferret model under certain conditions. These findings suggest that highly pathogenic influenza strains may have broader tropism and therefore may be transmitted by mechanisms other than the naso-pharyngeal route. Although our current study does not provide direct evidence of virus replication in blood, H5N1 replication in blood cells may warrant further exploration.

## Competing interests

The authors declare that there are no competing interests.

## Authors’ contributions

IH conceived of the study. XW, IH designed the experiments. XW, JT, JZ, ZY performed study and data analysis. XW and IH wrote the paper. The authors read and approved the final manuscript.

## Pre-publication history

The pre-publication history for this paper can be accessed here:

http://www.biomedcentral.com/1471-2334/14/192/prepub

## Supplementary Material

Additional file 1: Table S1 Hemagglutination inhibition assay with blood from ferrets challenged with H5N1 virus, A/VN/1203/04. **Figure S1.** Alignment and compare of partial sequence of wild type (infection) M (matrix) gene of H5N1 virus with these sequenced from bloods of some recipients. Click here for file
