# Supplementary material for: Highly pathogenic avian influenza A virus (H5N1) can be transmitted in ferrets by transfusion
Source: BMC Infect Dis. 2014 Apr 8;14:192. doi: 10.1186/1471-2334-14-192 (PMC4101865; doi:10.1186/1471-2334-14-192)
Supplement: Additional file 1: Table S1 — Hemagglutination inhibition assay with blood from ferrets challenged with H5N1 virus, A/VN/1203/04. Figure S1. Alignment and compare of partial sequence of wild type (infection) M (matrix) gene of H5N1 virus with these sequenced from bloods of some recipients. [file 1471-2334-14-192-S1.doc]

**Additional file 1**

**Hemagglutination inhibition (HAI) assay.**

Sera were treated with receptor-destroying enzyme (RDE) by diluting one part serum with three parts enzyme and incubated overnight in 37°C water bath. The enzyme was inactivated by 30 min. incubation at 56°C followed by addition of six parts PBS for a final dilution of 1/10. HAI assays were performed in V-bottom 96-well plates using four hemagglutinating units (HAU) of virus and 1% horse red blood cells (HRBC). Briefly, HRBCs were washed and resuspended to a final concentration of 1% (assuming that the packed HRBCs were at a concentration of 75%) in 1× PBS containing 0.5% bovine serum albumin (from fraction V; Sigma). The RDE-treated serum samples were serially diluted in PBS from 1:20 to 1:2,560. Serum and virus were incubated for 60 minutes at room temperature. After incubation, 50 μl of 1% HRBCs was added to every well. Plates were tapped to ensure mixing, covered, and incubated for 60 min at room temperature. Plates were then tilted and wells observed for agglutination or non-agglutination. A reference standard for A/VN/1203/04 which was generated in ferrets at the CDC was included as a positive control as part of each plate setup. Data were expressed as final titer of sample dilution.

**Table S1.** Hemagglutination inhibition assay with blood from ferrets challenged with H5N1 virus, A/VN/1203/04

| Infection dose | Group | Animal code | Titer | Day of blood | Day of viral  Load in blood |
| --- | --- | --- | --- | --- | --- |
| High dose | Donor | 58 | 640 | 8 |  |
|  | Recipients | 77 | 2560 | 14 |  |
|  |  | 79 | 1280 | 14 | 8 |
|  |  | 83 | 1280 | 14 |  |
|  |  | 86 | 1280 | 8 | 8 |

The Hemagglutination inhibition (HAI) assay tested all the blood samples of different time points from both donors and recipients. The positive results were in Supplemental Table.

**Figure S1.** Alignment and compare of partial sequence of wild type (infection) M (matrix) gene of H5N1 virus with these sequenced from bloods of some recipients.

8 recipient bloods were selected randomly. Virus RNAs were isolated, and then were RT-PCR and analyzed with sequencing.

Influenza A virus (A/Viet Nam/1203/2004(H5N1))matrix protein 1 (M) genes, complete cds length=982

Score = 1305 bits (1446), Expect = 0.0; Identities = 723/723 (100%), Gaps = 0/723 (0%)

Strand=Plus/Plus

Query 1 TCCTGTCACCTCTGACTAAAGGGATTTTGGGATTTGTATTCACGCTCACCGTGCCCAGTG 60

||||||||||||||||||||||||||||||||||||||||||||||||||||||||||||

Sbjct 152 TCCTGTCACCTCTGACTAAAGGGATTTTGGGATTTGTATTCACGCTCACCGTGCCCAGTG 211

Query 61 AGCGAGGACTGCAGCGTAGACGCTTTGTCCAGAATGCCCTAAATGGAAATGGAGATCCAA 120

||||||||||||||||||||||||||||||||||||||||||||||||||||||||||||

Sbjct 212 AGCGAGGACTGCAGCGTAGACGCTTTGTCCAGAATGCCCTAAATGGAAATGGAGATCCAA 271

Query 121 ATAATATGGATAGGGCAGTTAAGCTATATAAGAAGCTGAAAAGAGAAATAACATTCCATG 180

||||||||||||||||||||||||||||||||||||||||||||||||||||||||||||

Sbjct 272 ATAATATGGATAGGGCAGTTAAGCTATATAAGAAGCTGAAAAGAGAAATAACATTCCATG 331

Query 181 GGGCTAAGGAGGTCGCACTCAGCTACTCAACCGGTGCACTTGCCAGTTGCATGGGTCTCA 240

||||||||||||||||||||||||||||||||||||||||||||||||||||||||||||

Sbjct 332 GGGCTAAGGAGGTCGCACTCAGCTACTCAACCGGTGCACTTGCCAGTTGCATGGGTCTCA 391

Query 241 TATACAACAGGATGGGAACGGTGACTACGGAAGTGGCTTTTGGCCTAGTGTGTGCCACTT 300

||||||||||||||||||||||||||||||||||||||||||||||||||||||||||||

Sbjct 392 TATACAACAGGATGGGAACGGTGACTACGGAAGTGGCTTTTGGCCTAGTGTGTGCCACTT 451

Query 301 GTGAGCAGATTGCAGATTCACAGCATCGGTCTCACAGACAGATGGCAACTATCACCAACC 360

||||||||||||||||||||||||||||||||||||||||||||||||||||||||||||

Sbjct 452 GTGAGCAGATTGCAGATTCACAGCATCGGTCTCACAGACAGATGGCAACTATCACCAACC 511

Query 361 CACTAATCAGACATGAGAACAGAATGGTGCTGGCCAGCACTACAGCTAAGGCTATGGAGC 420

||||||||||||||||||||||||||||||||||||||||||||||||||||||||||||

Sbjct 512 CACTAATCAGACATGAGAACAGAATGGTGCTGGCCAGCACTACAGCTAAGGCTATGGAGC 571

Query 421 AGATGGCGGGATCAAGTGAGCAGGCAGCGGAAGCCATGGAGATCGCTAATCAGGCTAGGC 480

||||||||||||||||||||||||||||||||||||||||||||||||||||||||||||

Sbjct 572 AGATGGCGGGATCAAGTGAGCAGGCAGCGGAAGCCATGGAGATCGCTAATCAGGCTAGGC 631

Query 481 AGATGGTGCAGGCAATGAGGACAATTGGGACTCATCCTAACTCTAGTGCTGGTCTGAGAG 540

||||||||||||||||||||||||||||||||||||||||||||||||||||||||||||

Sbjct 632 AGATGGTGCAGGCAATGAGGACAATTGGGACTCATCCTAACTCTAGTGCTGGTCTGAGAG 691

Query 541 ATAATCTTCTTGAAAATTTGCAGGCCTACCAGAAACGAATGGGAGTGCAGATGCAGCGAT 600

||||||||||||||||||||||||||||||||||||||||||||||||||||||||||||

Sbjct 692 ATAATCTTCTTGAAAATTTGCAGGCCTACCAGAAACGAATGGGAGTGCAGATGCAGCGAT 751

Query 601 TCAAGTGATCCTATTGTTGTTGCCGCAAATATCATTGGGATCTTGCACTTGATATTGTGG 660

||||||||||||||||||||||||||||||||||||||||||||||||||||||||||||

Sbjct 752 TCAAGTGATCCTATTGTTGTTGCCGCAAATATCATTGGGATCTTGCACTTGATATTGTGG 811

Query 661 ATTCTTGATCGTCTTTTCTTCAAATGCATTTATCGTCGCCTTAAATACGGTTTGAAAAGA 720

||||||||||||||||||||||||||||||||||||||||||||||||||||||||||||

Sbjct 812 ATTCTTGATCGTCTTTTCTTCAAATGCATTTATCGTCGCCTTAAATACGGTTTGAAAAGA 871

Query 721 GGG 723

|||

Sbjct 872 GGG 874

Clone1

TCCTGTCACCTCTGACTAAAGGGATTTTGGGATTTGTATTCACGCTCACCGTGCCCAGTGAGCGAGGACTGCAGCGTAGACGCTTTGTCCAGAATGCCCTAAATGGAAATGGAGATCCAAATAATATGGATAGGGCAGTTAAGCTATATAAGAAGCTGAAAAGAGAAATAACATTCCATGGGGCTAAGGAGGTCGCACTCAGCTACTCAACCGGTGCACTTGCCAGTTGCATGGGTCTCATATACAACAGGATGGGAACGGTGACTACGGAAGTGGCTTTTGGCCTAGTGTGTGCCACTTGTGAGCAGATTGCAGATTCACAGCATCGGTCTCACAGACAGATGGCAACTATCACCAACCCACTAATCAGACATGAGAACAGAATGGTGCTGGCCAGCACTACAGCTAAGGCTATGGAGCAGATGGCGGGATCAAGTGAGCAGGCAGCGGAAGCCATGGAGATCGCTAATCAGGCTAGGCAGATGGTGCAGGCAATGAGGACAATTGGGACTCATCCTAACTCTAGTGCTGGTCTGAGAGATAATCTTCTTGAAAATTTGCAGGCCTACCAGAAACGAATGGGAGTGCAGATGCAGCGATTCAAGTGATCCTATTGTTGTTGCCGCAAATATCATTGGGATCTTGCACTTGATATTGTGGATTCTTGATCGTCTTTTCTTCAAATGCATTTATCGTCGCCTTAAATACGGTTTGAAAAGAGGG

Clone2

TCCTGTCACCTCTGACTAAAGGGATTTTGGGATTTGTATTCACGCTCACCGTGCCCAGTGAGCGAGGACTGCAGCGTAGACGCTTTGTCCAGAATGCCCTAAATGGAAATGGAGATCCAAATAATATGGATAGGGCAGTTAAGCTATATAAGAAGCTGAAAAGAGAAATAACATTCCATGGGGCTAAGGAGGTCGCACTCAGCTACTCAACCGGTGCACTTGCCAGTTGCATGGGTCTCATATACAACAGGATGGGAACGGTGACTACGGAAGTGGCTTTTGGCCTAGTGTGTGCCACTTGTGAGCAGATTGCAGATTCACAGCATCGGTCTCACAGACAGATGGCAACTATCACCAACCCACTAATCAGACATGAGAACAGAATGGTGCTGGCCAGCACTACAGCTAAGGCTATGGAGCAGATGGCGGGATCAAGTGAGCAGGCAGCGGAAGCCATGGAGATCGCTAATCAGGCTAGGCAGATGGTGCAGGCAATGAGGACAATTGGGACTCATCCTAACTCTAGTGCTGGTCTGAGAGATAATCTTCTTGAAAATTTGCAGGCCTACCAGAAACGAATGGGAGTGCAGATGCAGCGATTCAAGTGATCCTATTGTTGTTGCCGCAAATATCATTGGGATCTTGCACTTGATATTGTGGATTCTTGATCGTCTTTTCTTCAAATGCATTTATCGTCGCCTTAAATACGGTTTGAAAAGAGGG

Clone3

TCCTGTCACCTCTGACTAAAGGGATTTTGGGATTTGTATTCACGCTCACCGTGCCCAGTGAGCGAGGACTGCAGAGTAGACGCTTTGTCCAGAATGCCCTAAATGGAAATGGAGATCCAAATAATATGGATAGGGCAGTTAAGCTATATAAGAAGCTGAAAAGAGAAATAACATTCCATGGGGCTAAGGAGGTCGCACTCAGCTACTCAACCGGTGCACTTGCCAGTTGCATGGGTCTCATATACAACAGGATGGGAACGGTGACTACGGAAGTGGCTTTTGGCCTAGTGTGTGCCACTTGTGAGCAGATTGCAGATTCACAGCATCGGTCTCACAGACAGATGGCAACTATCACCAACCCACTAATCAGACATGAGAACAGAATGGTGCTGGCCAGCACTACAGCTAAGGCTATGGAGCAGATGGCGGGATCAAGTGAGCAGGCAGCGGAAGCCATGGAGATCGCTAATCAGGCTAGGCAGATGGTGCAGGCAATGAGGACAATTGGGACTCATCCTAACTCTAGTGCTGGTCTGAGAGATAATCTTCTTGAAAATTTGCAGGCCTACCAGAAACGAATGGGAGTGCAGATGCAGCGATTCAAGTGATCCTATTGTTGTTGCCGCAAATATCATTGGGATCTTGCACTTGATATTGTGGATTCTTGATCGTCTTTTCTTCAAATGCATTTATCGTCGCCTTAAATACGGTTTGAAAAGAGGG

Clone4

TCCTGTCACCTCTGACTAAAGGGATTTTGGGATTTGTATTCACGCTCACCGTGCCCAGTGAGCGAGGACTGCAGCGTAGACGCTTTGTCCAGAATGCCCTAAATGGAAATGGAGATCCAAATAATATGGATAGGGCAGTTAAGCTATATAAGAAGCTGAAAAGAGAAATAACATTCCATGGGGCTAAGGAGGTCGCACTCAGCTACTCAACCGGTGCACTTGCCAGTTGCATGGGTCTCATATACAACAGGATGGGAACGGTGACTACGGAAGTGGCTTTTGGCCTAGTGTGTGCCACTTGTGAGCAGATTGCAGATTCACAGCATCGGTCTCACAGACAGATGGCAACTATCACCAACCCACTAATCAGACATGAGAACAGAATGGTGCTGGCCAGCACTACAGCTAAGGCTATGGAGCAGATGGCGGGATCAAGTGAGCAGGCAGCGGAAGCCATGGAGATCGCTAATCAGGCTAGGCAGATGGTGCAGGCAATGAGGACAATTGGGACTCATCCTAACTCTAGTGCTGGTCTGAGAGATAATCTTCTTGAAAATTTGCAGGCCTACCAGAAACGAATGGGAGTGCAGATGCAGCGATTCAAGTGATCCTATTGTTGTTGCCGCAAATATCATTGGGATCTTGCACTTGATATTGTGGATTCTTGATCGTCTTTTCTTCAAATGCATTTATCGTCGCCTTAAATACGGTTTGAAAAGAGGG

Clone5

TCCTGTCACCTCTGACTAAAGGGATTTTGGGATTTGTATTCACGCTCACCGTGCCCAGTGAGCGAGGACTGCAGCGTAGACGCTTTGTCCAGAATGCCCTAAATGGAAATGGAGATCCAAATAATATGGATAGGGCAGTTAAGCTATATAAGAAGCTGAAAAGAGAAATAACATTCCATGGGGCTAAGGAGGTCGCACTCAGCTACTCAACCGGTGCACTTGCCAGTTGCATGGGTCTCATATACAACAGGATGGGAACGGTGACTACGGAAGTGGCTTTTGGCCTAGTGTGTGCCACTTGTGAGCAGATTGCAGATTCACAGCATCGGTCTCACAGACAGATGGCAACTATCACCAACCCACTAATCAGACATGAGAACAGAATGGTGCTGGCCAGCACTACAGCTAAGGCTATGGAGCAGATGGCGGGATCAAGTGAGCAGGCAGCGGAAGCCATGGAGATCGCTAATCAGGCTAGGCAGATGGTGCAGGCAATGAGGACAATTGGGACTCATCCTAACTCTAGTGCTGGTCTGAGAGATAATCTTCTTGAAAATTTGCAGGCCTACCAGAAACGAATGGGAGTGCAGATGCAGCGATTCAAGTGATCCTATTGTTGTTGCCGCAAATATCATTGGGATCTTGCACTTGATATTGTGGATTCTTGATCGTCTTTTCTTCAAATGCATTTATCGTCGCCTTAAATACGGTTTGAAAAGAGGG

Clone6

TCCTGTCACCTCTGACTAAAAGGGATTTTGGGAATTTGTATTCACGCTCACCGTGCCCAGTGAGCGAGGACTGCAGCGTAGACGCTTTGTCCAGAATGCCCTAAATGGAAAATGGAGATCCAAATAAATATGGATAGGGCAGTTAAGCTATATAAGAAGCTGAAAAGAGAAATAACATTCCATGGGGCTAAGGAGGTCGCACTCAGCTACTCAACCGGTGCACTTGCCAGTTGCATGGGTCTCATATACAACAGGATGGGAACGGTGACTACGGAAGTGGCTTTTGGCCTAGTGTGTGCCACTTGTGAGCAGATTGCAGATTCACAGCATCGGTCTCACAGACAGATGGCAACCATCACCAACCCACTAATCAGGCATGAGAACAGAATGGTGCTGGCCAGCACTACAGCTAAGGCTATGGAGCAGATGGCGGGATCAAGCGAGCAGGCAGCGGAAGCCATGGAAGTCGCTAATCAGGCTAGGCAGATGGTGCAGGCAATGAGGACAATTGGGACTCATCCTAACTCTAGTGCTGGTCTGAGAGATAATCTTCTTGAAAATTTGCAGGCCTACCAGAAACGAATGGGAGTGCAGATGCAGCGATTCAAGTGATCCTCTTGTTGTTGCCGCAAGTATCATTGGGATCTTGCACTTGATATTGTGGATTCTTGATCGTCTTTTCTTCAAATGCATTAATCGTCGCCTTAAATACGGTTTGAAAAGAGGG

Clone7

TCCTGTCACCTCTGACTAAAGGGATTTTGGGATTTGTATTCACGCTCACCGTGCCCAGTGAGCGAGGACTGCAGCGTAGACGCTTTGTCCAGAATGCCCTAAATGGAAATGGAGATCCAAATAATATGGATAGGGCAGTTAAGCTATATAAGAAGCTGAAAAGAGAAATAACATTCCATGGGGCTAAGGAGGTCGCACTCAGCTACTCAACCGGTGCACTTGCCAGTTGCATGGGTCTCATATACAACAGGATGGGAACGGTGACTACGGAAGTGGCTTTTGGCCTAGTGTGTGCCACTTGTGAGCAGATTGCAGATTCACAGCATCGGTCTCACAGACAGATGGCAACCATCACCAACCCACTAATCAGGCATGAGAACAGAATGGTGCTGGCCAGCACTACAGCTAAGGCTATGGAGCAGATGGCGGGATCAAGCGAGCAGGCAGCGGAAGCCATGGAAGTCGCTAATCAGGCTAGGCAGATGGTGCAGGCAATGAGGACAATTGGGACTCATCCTAACTCTAGTGCTGGTCTGAGAGATAATCTTCTTGAAAATTTGCAGGCCTACCAGAAACGAATGGGAGTGCAGATGCAGCGATTCAAGTGATCCTCTTGTTGTTGCCGCAAGTATCATTGGGATCTTGCACTTGATATTGTGGATTCTTGATCGTCTTTTCTTCAAATGCATTTATCGTCGCCTTAAATACGGTTTGAAAAGAGGG

Clone8

CCCCGTAACCTCTGACTAAAGGGATTTTGGGATTTGGATTCACGCTCACCGTGCCCAATGAGCGAGGACTGCAGCGTAAACGCTTTGTCCAGAATGCCCTAAATGGAAATGGAGATCCAAATAATATGGATAGGGCAGTTAAGCGATATAAGAAGCTGAAAAGAGAAATAACATTCCATGGGGCTAAGGAGGTCGCACTCAGCTACTCAACCGGTGCACTTGCCAGTTGCATGGGTCTCAAATACAACAGGATGGGAACGGTGACTACGGAACTGGCTTTTGGCCTAGTGTGTGCCACTTGTGAGCAGATTGCAGATTCACAGCATCGGTCTCACAGACAGATGGCAACTATCACCAACCCACTAATCAGACATGAGAACAGAATGGTGCTGGCCAGCACTACAGCTAAGGCTATGGAGCAGATGGCGGGATCAAGTGAGCAGGCAGCGGAAGCCATGGAGATCGCTAATCAGGCTAGGCAGATGGTGCAGGCAATGAGGACAATTGGGACTCATCCTAACTCTAGTGCTGGTCCGAGAGATAATCTTCTTGAAAATTTGCAGGCCTACCAGAAACGAATGGGAGTGCAGATGCACAGATTCAAGTGATCCAAACCAACTAGCCGCAAATATCATTGGGGATCTTGCACTTGATATTGTGGATTCTTGATCGTCTTTTCTTCAAATGCATTTATCGGTCGCCTTAAATACGGTTTGAAAAGAGGG

As shown in Supplemental Figure, almost exact M1 gene sequence was detected from virus H5N1 (A/NT/1203/2004) in the blood of recipient animals; suggesting that virus H5N1 was able to be transmitted through blood transfusion without affecting the virus gene sequences.
